# Influence of poly(*N*-isopropylacrylamide) (PIPAAm) graft density on properties of PIPAAm grafted poly(dimethylsiloxane) surfaces and their stability

**DOI:** 10.1016/j.heliyon.2021.e06520

**Published:** 2021-03-16

**Authors:** Yoshikatsu Akiyama

**Affiliations:** Institute of Advanced Biomedical Engineering and Science, Tokyo Women's Medical University (TWIns), 8-1 Kawada-cho, Shinjuku-ku, Tokyo 162-8886, Japan

**Keywords:** Electron beam irradiation, Temperature-responsive cell culture surface, Mechanical stress, poly(*N*-isopropylacrylamide), Polydimethylsiloxane

## Abstract

A previous report shows that poly(*N*-isopropylacrylamide) (PIPAAm) gel grafted onto poly(dimethylsiloxane) (PDMS) (PI-PDMS) surfaces with large PIPAAm graft density (Lar-PI-PDMS), is prepared by using electron beam irradiation, demonstrating that applied mechanical stretching affects properties of the Lar-PI-PDMS surface. However, the influence of PIPAAm graft density on the properties of PI-PDMS surfaces and their stability are not understood. To provide insight into these points, the properties of PI-PDMS surfaces with low PIPAAm graft density (Low-PI-PDMS) surfaces with stretched (stretch ratio = 20%) and unstretched states were examined as stretchable temperature-responsive cell culture surface using contact angle measurement and cell attachment/detachment assays, compared to those with Lar-PI-PDMS, as previously reported. Long-term contact angle measurements (61 days) for unstretched Low-PI-PDMS and Lar-PI-PDMS surfaces indicated that the cross-linked structure of the grafted PIPAAm gel suppressed hydrophobic recovery of the basal PDMS surface. The cell attachment assay revealed that the stretched Low-PI-PDMS surface was less cell adhesive than that of the unstretched Low-PI-PDMS surface despite of a larger amount of adsorbed fibronectin (FN). The lower cell adhesiveness was possibly explained by denaturation of adsorbed FN, which was induced by the strong hydrophobic property of the stretched Low-PI-PDMS surface. The cell detachment assay revealed that dual stimuli, low temperature treatment and mechanical shrinking stress applied to the stretched Low-PI-PDMS surface promoted cell detachment compared to a single stimulus, low temperature treatment or mechanical shrinking stress. These results suggested that the PIPAAm gelgrafted PDMS surface was chemically stable and did not suffer from hydrophobic recovery. External mechanical stretching stress not only strongly dehydrated grafted PIPAAm chains, but also denatured the adsorbed FN when the grafted PIPAAm layer was extremely thin, as in Low-PI-PDMS surfaces. Thus, PI-PDMS may be utilized as a stretchable temperature-responsive cell culture surface without significant hydrophobic recovery.

## 1 Introduction

A temperature-responsive cell culture surface (TRCS), which was first invented by Okano *et al.*, has been a powerful tool for the fabrication of cell-sheets [1, 2]. They demonstrated that cell-sheets are transplantable to damaged human tissues and organs to helptheir recovery, while thick tissue with high cell density (*e.g*., heart muscle) was able to be fabricated by repeatedly stacking cell-sheets [3, 4, 5]. So far, the damaged cornea, heart, periodontal ligament tissue, esophagus, cartilage, and middle ear have been successfully treated using transplantation of cell-sheets [5].

A TRCS has been prepared by grafting poly(*N*-isopropylacrylamide) (PIPAAm) gel onto tissue culture polystyrene (PI-TCPS) or a glass surface (PI-GS) using electron beam (EB) irradiation [2, 6, 7]. Akiyama *et al.* have demonstrated that precise control of the thickness of the nanoscale grafted PIPAAm layer is a key factor for TRCSs to show temperature-dependent cell attachment and detachment character [3]. PI-TCPS and PI-GS with more than 30 nm and 5 nm of grafted PIPAAm gel layer, respectively, are so hydrophilic that cells fail to adhere even at 37 °C, where the grafted PIPAAm chains are dehydrated, while these surfaces with optimized thickness (20 nm and 4.8 nm for PI-TCPS and PI-GS, respectively) are able to exhibit temperature-dependent cell attachment and detachment characteristics [7, 8]. The polymer-thickness dependency on cell adhesive and hydrophobic properties of these TRCSs has been previously explained in terms of different molecular mobilities of the grafted PIPAAm chains between thinner and thicker PIPAAm layers as follows [3, 6, 7, 8]. Grafted PIPAAm chains are strongly dehydrated and dynamically restricted at the interface of the basal surface of tissue culture polystyrene or glass [3, 6, 7, 8]. In the case of a thinner PIPAAm grafted gel layer (the optimized thickness of the grafted PIPAAm gel layer as mentioned above), such restriction and dehydration progressively promotes dehydration of the grafted PIPAAm chains at the outermost region of PI-TCPS and PI-GS [6, 7]. Consequently, PI-TCPS and PI-GS with thinner PIPAAm grafted gel layer shows cell adhesive character, because PIPAAm chains at the outermost region are so dehydrated [6, 7]. However, when the grafted PIPAAm gel is thicker, such restriction and dehydration are not able to dehydrate the PIPAAm chains at the outermost region [6, 7]. Therefore, the grafted PIPAAm chains are not so dehydrated that cells do not adhere even at 37 °C.

Based on the graft-polymer thickness dependency on the properties of subsequent TRCSs, to readily modulate the thickness of the grafted PIPAAm gel layer as well as properties of TRCSs, a stretchable temperature-responsive cell culture surface has been developed: a PIPAAm gel grafted polydimethylsiloxane (PDMS) (PI-PDMS) surface with a large amount of grafted PIPAAm (Lar-PI-PDMS), using EB irradiation [9]. The external mechanical stretching stress enables a decrease in the thickness of the grafted PIPAAm layer of Lar-PI-PDMS, enhancing the hydrophobic and cell adhesive properties of Lar-PI-PDMS [9]. This is because, by thinning the grafted PIPAAm gel layer with external mechanical stress, the grafted PIPAAm chains at the outermost surface of Lar-PI-PDMS are dehydrated [9]. After culturing cells on stretched Lar-PI-PDMS surfaces, successive application of low temperature stimulus and mechanical shrinking stress to stretched Lar-PI-PDMS allowed for more rapid cell detachment and cell sheet recovery than single stimulus, either low temperature or mechanical shrining stress. Shrinking stretched Lar-PI-PDMS (releasing the stretched stress) enabled an increase in the thickness of the grafted PIPAAm layer, promoting hydration of grafted PIPAAm chains at the outermost surface of Lar-PI-PDMS [9]. That is to say, hydration of the grafted PIPAAm chains was more promoted by dual stimuli; mechanical shrinking stress and low temperature treatment.

These results demonsrated that the external mechanical stress altered properties of PI-PDMS surfaces. However, it is unclear whether external mechanical stress is available, or not, to control the properties of PI-PDMS surface when PIPAAm grafted density (Low-PI-PDMS (PI-PDMS with lower PIPAAm grafted density)) is lower than that of Lar-PI-PDMS.

In contrast, PDMS has been exploited as a biomaterial surface owing to its low cost, oxygen permeability, unique stretchability, and ease of micro-patenting [10, 11, 12, 13]. To improve the biocompatibility of hydrophobic PDMS surfaces, plasma treatment or grafting hydrophilic moieties, such as poly(ethylene glycol) and sulfobetaine has been employed to render PDMS surfaces hydrophilic [14, 15, 16, 17, 18, 19]. However, the hydrophilic PDMS surfaces gradually recover hydrophobic properties or return to the original hydrophobic PDMS over time, because of the hydrophobic recovery accompanying condensation of surface silanol groups and transport of low-molecular-mass to PDMS surface [14, 15, 16, 17, 18, 19, 20, 21]. To the best of my knowledge, there have been no reports on the long-term stability of the PI-PDMS surface. Information on the stability of the PI-PDMS surface is important to utilize the PI-PDMS surface as a cell culture surface.

In this study, to examine the stability of Lar-PI-PDMS and Low-PI-PDMS surfaces, the contact angles of these two PI-PDMS surfaces were measured at appropriate intervals up to 61 days below and above the lower critical solution temperature. To investigate the influence of grafted PIPAAm density on the properties of PI-PDMS surfaces, cell attachment and detachment behaviors of the Low-PI-PDMS surface has been compared to those of Lar-PI-PDMS surfaces as reported previously [9]. Fibronectin (FN) adsorption experiments were also performed to investigate how external mechanical stretching and/or shrinking stresses affected FN adsorption on Lar-PI-PDMS and Low-PI-PDMS surfaces. In conclusion, these PI-PDMS surfaces were not likely to suffer from the hydrophobic recovery of the basal PDMS. The external mechanical stretching stress altered Low-PI-PDMS surface to be so hydrophobic that the adsorbed FN was denatured. Therefore, the stretched Low-PI-PDMS surfaces were less cell adhesive than that of the unstretched Low-PI-PDMS surfaces. In addition, dual stimuli, low temperature treatment and mechanical shrinking stress, promoted cell detachment from the stretched Low-PI-PDMS surface compared to a single stimulus (low temperature and/or mechanical shrinking stress). These results suggested PI-PDMS surfaces were utilized as stretchable temperature-responsive cell culture surface. Moreover, design and concept of the dual-stimuli responsive materials, PI-PDMS, may be available for the preparation of multi-component stimuli-responsive materials based on the PIPAAm component [22, 23].

## 2 Materials and methods

### 2.1 Preparation of the poly(*N*-isopropylacryylamide) gel grafted PDMS surface

Residual uncrosslinked PDMS and catalytic components in PDMS cell culture chamber (4-well type, STREX (Osaka, Japan)) were extracted with sequential immersion of triethylamine, ethyl acetate, and acetone solvents [24]. The resultant PDMS surface was subjected to O_2_ plasma (30 W, 80 m Torr O_2_, 10 s) to form silanol groups. Immediately, the plasma-treated PDMS surfaces were reacted in a 1 wt% (v/v) aqueous solution of 3-aminopropyltrimethoxysilane (200 mL) to immobilize aminopropyl groups at 70 °C for 30 min (abbreviated as AP-PDMS) according to a previous report [25]. The resultant AP-PDMS surface was washed with water, and completely dried with an air spray gun. Immediately after drying, 15 μL of a 40 wt% or 30 wt% IPAAm monomer solution in a 2-propanol and methanol mixture (1:1 volume ratio) was dropped and spread onto each of the 4-wells of the AP-PDMS chamber. The PDMS substrate with the IPAAm monomer solution was subjected to EB irradiation to immobilize an ultrathin PIPAAm gel layer on the surface. EB irradiation conditions are the same in a previous report [9]. After removing unreacted monomer and ungrafted polymer by washing, the PIPAAm gel-grafted PDMS surface (PI-PDMS) was dried at 45 °C overnight and used for subsequent experiments. As described below, PI-PDMS surfaces with two different amounts of grafted PIPAAm gel were prepared.

### 2.2 Attenuated total reflection (ATR)/Fourier-transform infrared (FT-IR) measurements for determination of PIPAAm graft density of the PI-PDMS surface

One well of the PI-PDMS chamber was cut out (approximately 1.0 cm square) and used for determining the polymer graft density by FT-IR spectra using an a ATR method (NICOLET 6700, Thermo Scientific, MA, USA). The peak intensities at 1650 cm^−1^ and 1019 cm^−1^ (*I*1650, *I*1019) were measured, and the ratio of *I*1650/*I*1019 was calculated to determine the grafted PIPAAm density by referring to a calibration line in a previous report [9].

A calibration line was prepared as follows: an appropriate amount of commercially available PIPAAm (SIGMA-Aldrich, MO, USA) dissolved in 2-propanol and methanol solution (1:1 volume ratio) was cast on an AP-PDMS surface to prepare PIPAAm-coated PDMSs of known amounts. The PIPAAm-coated PDMSs were dried at room temperature for 12 h, and peak intensities at 1650 cm^−1^ and 1019 cm^−1^ were measured by ATR/FT-IR. The ratio of *I*1650/*I*1019 was plotted against the known amount of PIPAAm per square area of the AP-PDMS surface (1.0 cm^2^). The calibration line is indicated in a previous report [9].

PIPAAm graft density between 9.1 μg/cm^2^ and 14.7 μg/cm^2^ was selectively used for subsequent experiments as the PI-PDMS substrate with large PIPAA graft density, which was abbreviated as Lar-PI-PDMS. Lar-PI-PDMS was used for ATR/FT-IR, contact angle measurement and protein adsorption experiments, as described below. PI-PDMS with a low PIPAAm graft density from 6.9 μg/cm^2^ to 8.5 μg/cm^2^ was abbreviated as Low-PI-PDMS.

### 2.3 Contact angle measurements

Static contact angles were measured using the sessile drop method for PDMS, Low- and Lar-PI-PDMSs surfaces in both the unstretched and stretched states. Stretched states were created by applying 20% uniaxial stretching, as described below. Prior to the measurements, the lateral wall of the PDMS chamber or the PI-PDMS chamber was partially punched out to make a hole for observing a 2 μL water droplet, as previously reported [9]. Humidity was maintained between 40% and 60%, when the static contact angle measurement was conducted. The elastic substrates were uniaxially stretched using a stretching device (STREX, Osaka, Japan). The contact angle of a water droplet was measured perpendicular to the stretching direction and was determined using the *θ/2* method with a contact angle meter (DSA100, DAS3 software, KRÜSS GmbH, Hamburg, Germany). The contact angle measurement was conducted on appropriate days over a two-month period after obtaining PDMS, Lar-PI-PDMS and Low-PI-PDMS surfaces. The same PDMS, Lar- and Low-PI-PDMSs surfaces were repeatedly used for the contact angle measurements. After conducting each measurement, PDMS, Lar-, and Low-PI-PDMSs were lightly washed with Milli Q water, dried at 45 °C, and stored in a clean plastic box until the subsequent contact angle measurement.

The stretch ratio (SR) was defined according to a following equation;

SR (%) = (*L*_*x*_ – *L*_*0*_) / *L*_*0*_ × 100

where *L*_*x*_ and *L*_*0*_ represent the lateral lengths of the uniaxially stretched and unstrethed cell culture chambers, respectively. The SR = 20% condition was used for stretched samples. Three wells in a cell culture chamber were used for the contact angle measurements. Contact angles of more than five different places on the chamber of the unstretched and stretched PI-PDMS surfaces were measured. The contact angles are represented as the mean ± standard deviation. Student's t-test was used to determine differences in these contact angle values between stretched and unstretched states using Microsoft Excel (Microsoft Corporation. WA, USA). Contact angles measured long-term were represented as the mean ± standard deviation. In the same way, contact angles of a TCPS surface (FALCON (#353001), Corning Inc., NY, USA) and a bacteriological grade polystyrene (BPS, FALCON (#351008)) surfaces were measured at 37 °C.

### 2.4 Adsorption of FN on PDMS, stretched and unstretched PI-PDMSs

FN labeled with rhodamine (20 μg, Bovine plasma, Cytoskelton Inc., CO, USA) was dissolved in Dulbecco's phosphate buffered saline (D-PBS, Mg^2+^ and Ca^2+^ free) (Nacalai Tesque, Kyoto, Japan) and the concentration of the fluorescent FN was adjusted to 8 μg/ml for subsequent FN adsorption experiments. The FN solution (800 μl) was added to three wells of each PDMS, Lar- and Low-PI-PDMS chambers with unstretched and stretched states followed by incubation at 37 °C or 20 °C for 20 h (n = 3). After incubation, FN solution (150 μl) was taken from the well followed by added to a well in a 96-well microplate (bottom clear type (655096)), Greiner Bio-One International GmbH, Kuremensmünster, Australia) (n = 2). The fluorescent intensity of the FN solution was measured using a plate reader (SpectraMax M2, Molecular Devices, LLC., CA, USA)). A filter for 540 nm excitation and 580 nm emission was used for the detection of the rhodamine-labeled FN. The amount of adsorbed FN was calculated from the difference in the fluorescence intensity of FN solutions before and after the incubation. The amount of adsorbed FN is shown as the mean value with standard deviation.

### 2.5 Cell attachment/detachment assays with temperature and/or mechanical stimuli

**Figure 1:**
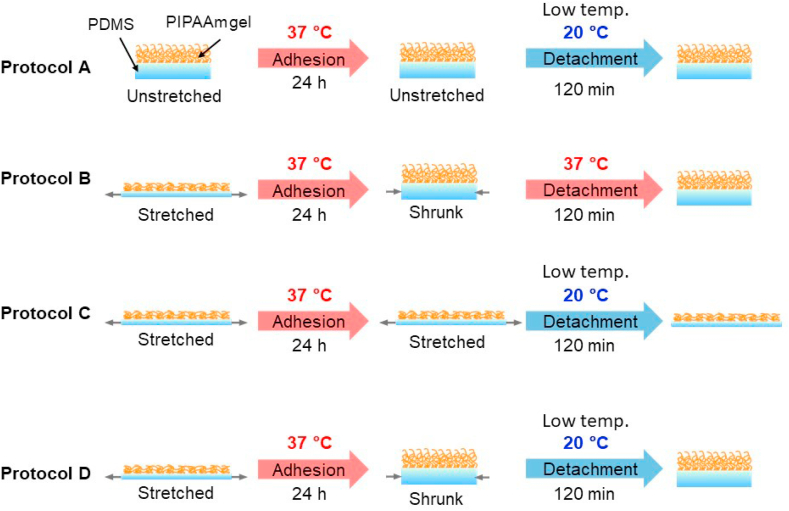
Illustration of four different protocols for cell attachment and detachment assay. These protocols were almost same as the cell detachment and attachment assay for Lar-PI-PDMS surface conducted in a previous report [9]. Protocol A: In the cell attachment assay, cells are seeded and cultured on unstretched Low-PI-PDMS surface. After culturing cells at 37 °C for 24 h, temperature is lowered to 20 °C for subsequent cell detachment assay. Protocol B and Protocol C: In the cell attachment assay, cells are seeded and cultured on stretched Low-PI-PDMS surfaces. After culturing cells at 37 °C for 24 h, single stimulus; either mechanical shrinking stress (Protocol B) or lowering temperature (Protocol C), is applied to the stretched PI-PDMS surfaces for the cell detachment assay. Protocol D: After culturing cells at 37 °C for 24 h, dual stimuli; lowering temperature and mechanical shrinking stress, are applied to stretched Low-PI-PDMS surface. Initial seeding BAECs density was 5.0 × 10^3^ cells/cm^2^. The numbers of adhered cells are counted using phase-contrast microscopic images, which were obtained at appropriate intervals during the cell attachment and detachment assay.

The cell attachment/detachment assay was performed using three wells of Low-PI-PDMS chamber. The Low-PI-PDMS chamber was stretched with a stretching device (purchased from STREX, Osaka, Japan), and was laid on a polystyrene (PSt) cell culture dish (150 mm (outer diameter), AGC technology, Shizuoka, Japan). The unstretched Low-PI-PDMS chamber was also laid on a PSt cell culture dish for the cell attachment/detachment assay. Cell culture samples were exposed to UV light for sterilization (5 min) prior to seeding the cells on the Low-PI-PDMS surfaces. Bovine aortic endothelial cells (BAECs, National Institute of Biomedical Innovation, Health and Nutrition, JCRB Cell Bank, Kobe, Japan, passage number <30) were seeded at 5.0 × 10^3^ cells/cm^2^ and cultured at 37 °C in a humidified atmosphere with 5% CO_2_ for 24 h. The number of adhered cells was counted using phase-contrast microscopic images, which were obtained at appropriate intervals up to 24 h. Four different procedures (Protocol A, B, C and D), as illustrated in Figure 1, were used for cell attachment and detachment assays, by using low temperature treatment and/or release of the mechanical stretching (shrinking the stretched Low-PI-PDMS). The cell attachment and detachment assay was conducted by refering a previous report [9]. In the cell attachment assay, cells were seeded on unstretched Low-PI-PDMS surfaces for Protocol A, and were seeded on stretched Low-PI-PDMS surfaces for Protocols B, C and D. Cells were cultured on these unstretched and stretched Low-PI-PDMS surfaces to investigate how the applied mechanical stress affected subsequent cell adhesiveness. After 24 h of cell culture, a cell detachment assay was successively conducted (Figure 1). In Protocol A, a low temperature stimulus was applied to unstretched Low-PI-PDMS surfaces. A single stimulus; either shrinking mechanical stress (Protocol B) or low-temperature treatment (Protocol C) was applied to the stretched Low-PI-PDMS surface. For Protocol D, dual stimuli, low-temperature treatment and mechanical shrinking stress, were successively applied to stretch the PI-PDMS surface.

During the cell detachment assay, microscopic images were taken with a digital camera at appropriate time intervals from 0 to 120 min, and the numbers of adhered cells was counted in the same way as described above. These values are shown as the mean with standard deviation. The cell attachment and detachment characteristics were discussed by comparing Lar-PI-PDMS in a previous report [9].

## 3 Results and discussion

**Figure 2:**
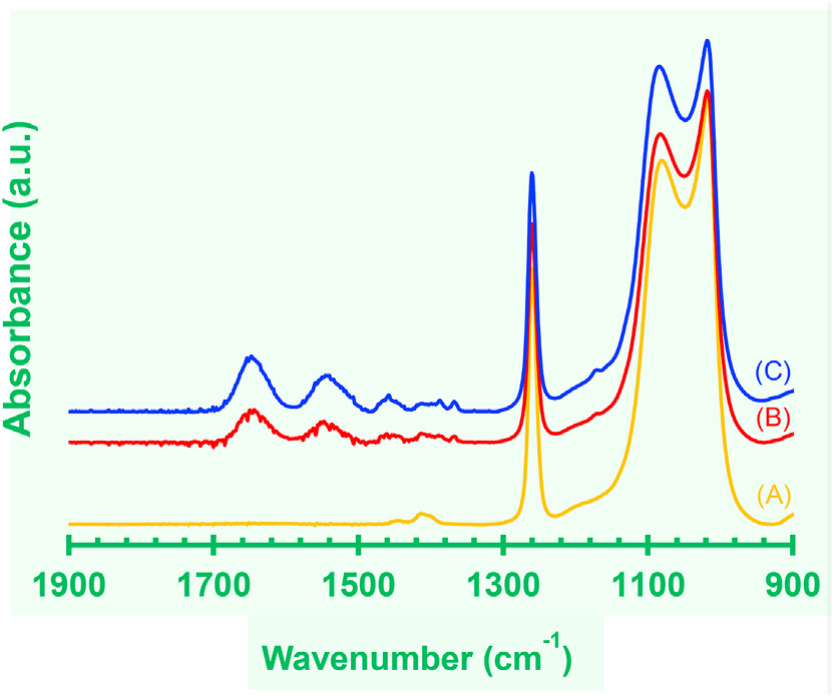
ATR/FT-IR spectra of (A) PDMS (yellow), (B) Low-PI-PDMS (red), and (C) Lar-PI-PDMS (blue) surfaces. Unstretched state is used for the measurement.

The ATR/FT-IR spectra of bare PDMS, Low-PI-PDMS and Lar-PI-PDMS surfaces are shown in Figure 2. Low-PI-PDMS and Lar-PI-PDMS surfaces were prepared using lower (30 wt%, Figure 2(B)) and higher (40 wt%, Figure 2(C)) IPAAm concentration, respectively. The spectrum of the Lar-PI-PDMS surface was nearly the same as the PIPAAm grafted PDMS surface in a previous report [9]. As described before, the grafted PIPAAm layer was deposited as a PIPAAm gel, because polymerization, cross-linking and grafting polymer proceed at the same time during EB irradiation [9]. The bare PDMS surface showed two broad signals and one sharp signal at 1019 cm^−1^, 1081 cm^−1^, and 1260 cm^−1^, which were assigned to siloxane bonding (Si–O–Si) and the methyl groups of PDMS, respectively (Figure 2(A)). In contrast, in the case of Lar-PI-PDMS and Low-PI-PDMS, two new signals emerged at 1650 cm^−1^ and 1549 cm^−1^, which were assigned to the C=O (amide I) and N–H (amide II) groups of grafted PIPAAm, respectively. The grafted PIPAAm density calculated from the peak intensity ratio indicated that Lar-PI-PDMS tended to show a larger PIPAAm graft density (from 9.1 μg/cm^2^ to 14.7 μg/cm^2^) (Figure 2(C)) than Low-PI-PDMS surface (from 6.9 μg/cm^2^ to 8.5 μg/cm^2^) (Figure 2(B)). The resultant PIPAAm graft density was controllable by the initial monomer concentration [9].

**Figure 3:**
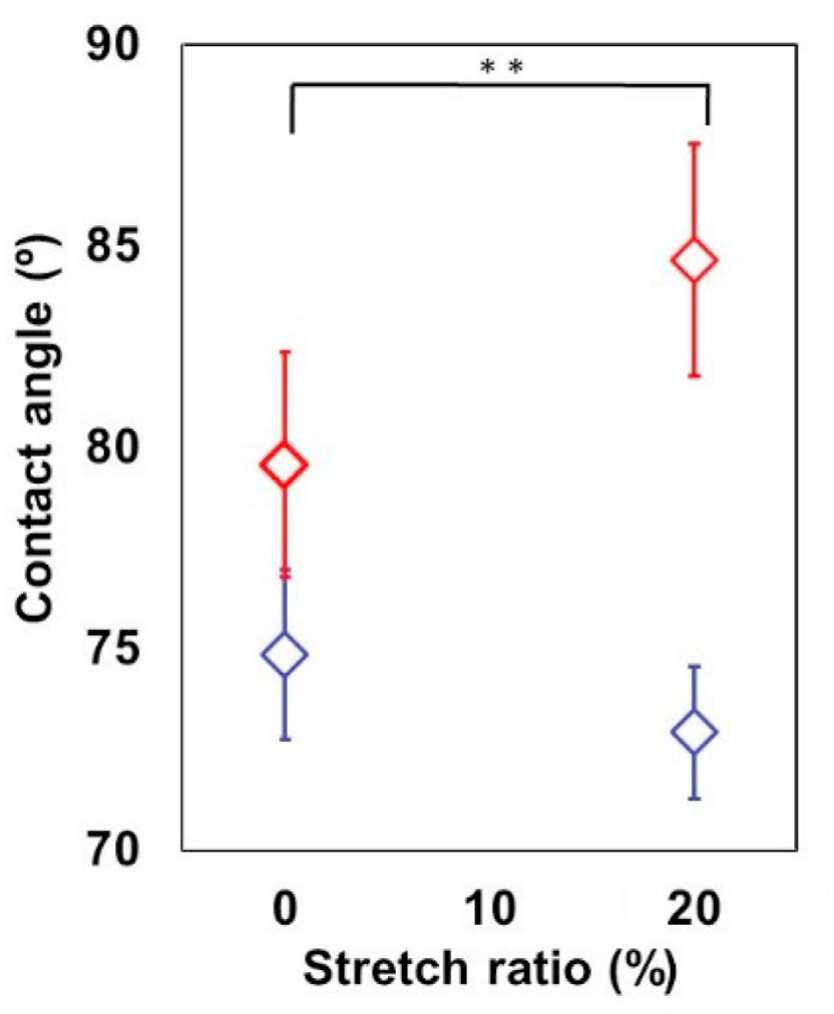
Contact angles of the unstretched and stretched Low-PI-PDMS (blue and red diamonds) at 20 °C (blue) and 37 °C (red). The stretch ratio (SR) of 20% is used for the stretched Low-PI-PDMS surfaces. Student's *t* test indicates statistically significant difference between stretched and unstretched Low-PI-PDMS at 37 °C (red diamond, *∗∗p* < 0.01). Statistically difference between stretched and unstretched Low-PI-PDMS is not observed at 20 °C (blue diamond).

Figure 3 shows contact angles of the unstretched and stretched Low-PI-PDMS surfaces. For comparison, contact angles of Lar-PI-PDMS surfaces were also measured (data not shown in Figure 3). The contact angles of the unstretched Lar-PI-PDMS surfaces decreased from 75.5° ± 1.3° (37 °C) to 66.1° ± 2.6° (20 °C) with low temperature treatment. By applying external uniaxial mechanical stress (SR = 20%), the contact angles of the unstretched Lar-PI-PDMS (75.5° ± 1.3° (37 °C) and 66.1° ± 2.6° (20 °C)) increased to 77.0 ± 1.9° (37 °C) and 73.2 ± 2.4° (20 °C). This is because, as reported previously [9], the applied mechanical stress reduces the thickness of the grafted PIPAAm gel layer, consequently, restricting the degree of mobility of grafted PIPAAm chains and promoting their dehydration.

External mechanical stress increased the contact angle in the Low-PI-PDMS surface at 37 °C (Figure 3). The contact angles of the unstretched Low-PI-PDMS surfaces (SR = 0%) were 79.6° ± 2.8° (red diamond, 37 °C) and 74.9° ± 2.1° (blue diamond, 20 °C), while those of stretched Low-PI-PDMS surfaces (SR = 20%) were 84.7 ± 2.9° (red diamond, 37 °C) and 72.3 ± 1.6° (blue diamond, 20 °C). Temperature-dependent contact angle changes were also observed for Low-PI-PDMS surfaces. The fact that the contact angles of the Low-PI-PDMS surfaces are larger than those of Lar-PI-PDMS is also explained by previous reports showing that lower PIPAAm graft density renders the grafted PIPAAm chains more dehydrated and dynamically restricted [6, 7]. The applied mechanical stress increased the contact angle of Low-PI-PDMS at 37 °C (red diamonds), whereas a statistically significant difference between stretched and unstretched Low-PI-PDMS was not observed at 20 °C (blue diamonds). The result might reflect the hypothesis that, when the grafted PIPAAm layer was extremely thin (as Low-PI-PDMS), external mechanical stress would not further restrict the molecular mobility and promote dehydration of the grafted PIPAAm chains at 20 °C.

**Figure 4:**
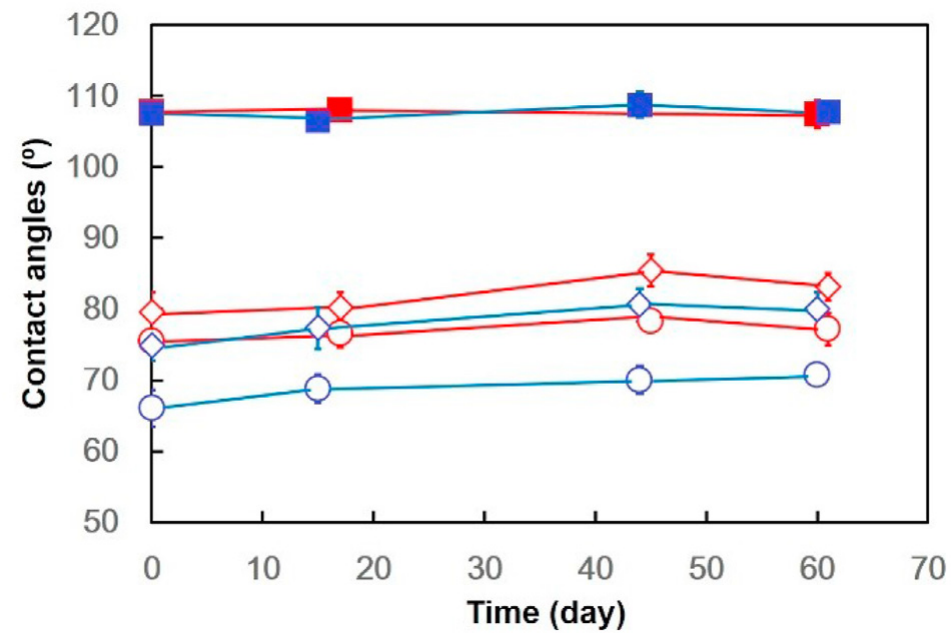
Time dependent contact angle values of unstretched PDMS (red and blue squares), unstretched Low-PI-PDMS (red and blue diamonds), and unstretched Lar-PI-PDMS (red and blue circles). Blue and red colors indicate the measurement temperature of contact angle values, 20 °C and 37 °C, respectively. The contact angles of the Low-PI-PDMS surface at day 0 are same as shown in Figure 3.

Plasma-treatment alters hydrophobic PDMS surfaces to be hydrophilic, however, the hydrophilic property returns to its original hydrophobic PDMS state within minutes to several hours after the plasma-treatment [20, 21]. This phenomenon is known as hydrophobic recovery, which is caused by the condensation of surface silanol groups and transport of non-cross-linked siloxane oligomers to the surface [20, 21]. Contact angle measurements have been frequently used to investigate hydrophobic recovery [14, 15, 16, 17, 18, 19]. Previous reports have demonstrated that the hydrophobic PDMS surface is hydrophilic at the time of grafting hydrophilic polymer (e.g., poly(ethylene glycol) (PEG) derivatives or poly(dimethylaminoethyl methacrylate)) to the PDMS surface. However, the hydrophilic surface gradually becomes hydrophobic again over time at ambient temperature owing to hydrophobic recovery [14, 15, 16, 17, 18, 19]. To investigate whether the properties of the PIPAAm-grafted PDMS surfaces were subjected to hydrophobic recovery, the contact angles of PDMS, Low-PI-PDMS, and Lar-PI-PDMS surfaces were measured at appropriate intervals up to 61 days (Figure 4). Temperature-dependent contact angles of the unstretched Lar-PI-PDMS and Low-PI-PDMS surfaces were observed, even after 61 days, although a very slight increase in contact angles was observed at 37 °C and 20 °C during the measurement term (from day 0 to day 61), while the contact angles of the PDMS surfaces were between 106.5° ± 1.0° and 107.9° ± 1.5°.

Maintaining the temperature-dependent contact angle change for the long-term (61 days) suggested that the hydrophobic recovery of the basal PDMS surface was strongly suppressed. The cross-linking structure of the grafted PIPAAm gel likely suppressed the dynamic transport of low-molecular-weight silicone compound, which induces hydrophobic recovery, suggesting that PI-PDMS surfaces may be chemically stable for the long term without suffering from hydrophobic recovery.

**Figure 5:**
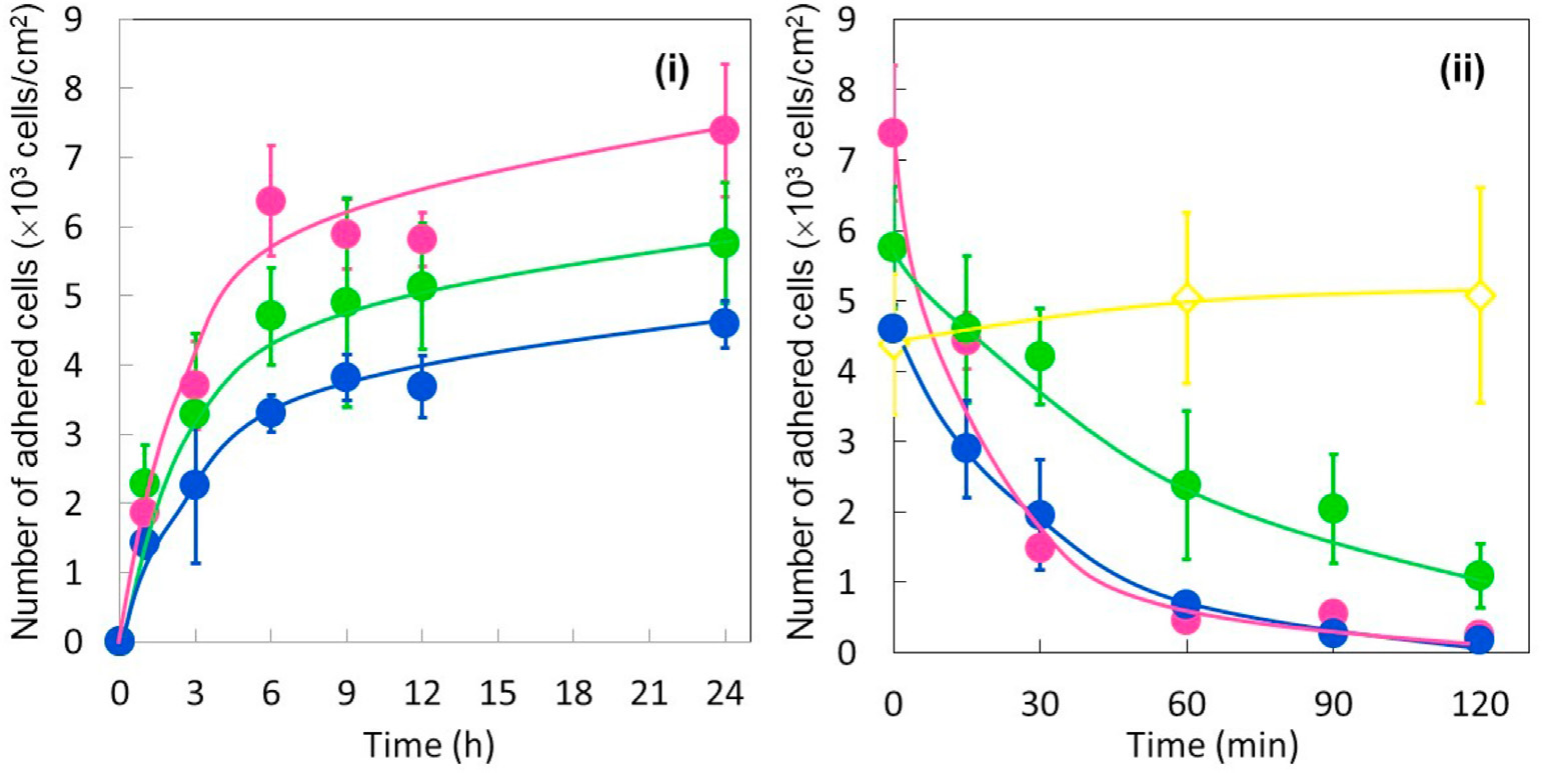
Cell attachment and detachment assay for stretched and unstretched Low-PI-PDMS surfaces ((i) cell attachment and (ii) cell detachment) with four different Protocols A, B, C, and D, as illustrated in Figure 1. For cell attachment assays, BAECs are seeded onto three stretched Low-PI-PDMS chambers and an unstretched chamber at a cell density of 5.0 × 10^3^ cells/cm^2^ at 37 °C. Each chamber consists of three Low-PI-PDMS wells for cell culture. The number of adhered cells is counted at appropriate intervals up to 24 h and plotted on the graph. (i) Cell attachment profiles are represented as green and blue circles (stretched Low-PI-PDMS surface), and pink circles (unstretched Low-PI-PDMS surface), respectively. (ii) After 24 h of cell culture, a cell detachment assay is conducted. Cell detachment profile of three stretched and one unstretched Low-PI-PDMS chamber using four different protocols are indicated as pink circles (Protocol A), yellow circles (Protocol B), green circles (Protocol C), and blue circles (Protocol D). Details of each protocols were described in the experimental section and illustrated in Figure 1.

**Figure 6:**
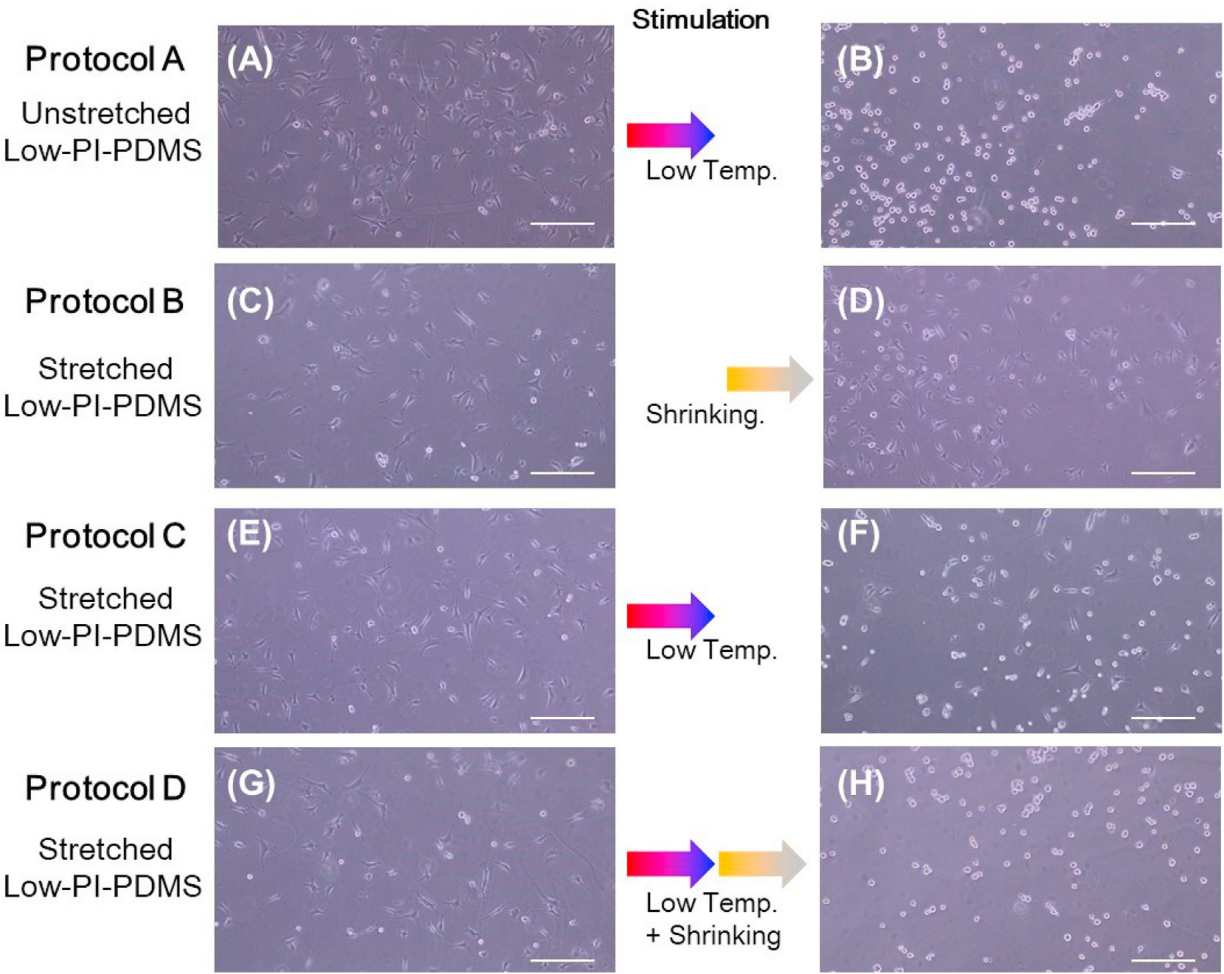
Phase-contrast microscopic images of cells on stretched and unstretched Low-PI-PDMS surfaces before and after cell detachment assay. (Left column) BAECs cultured on (A) unstretched and (C), (E), (G) stretched Low-PI-PDMS surfaces are shown at 24 h after seeding the cells at 37 °C before the cell detachment assay. (Right column) Cell detachment assay for (B) unstretched and (D), (F), (H) stretched Low-PI-PDMS surfaces after culturing cells for 24 h at 37 °C using four different protocols as illustrated in Figure 1. Photographs are taken at 90 min (B), (F), (H) and 120 min (D) after cell detachment assay is initiated. Protocol A: low temperature stimulus applied to an unstrethced Low-PI-PDMS surface (B). Protocol B: mechanical shrinking stimulus applied to a stretched Low-PI-PDMS surface (D). Protocol C: low temperature stimulus applied to a stretched Low-PI-PDMS surface (F). Protocol D: low temperature and mechanical shrinking stimuli applied to a stretched Low-PI-PDMS surface (H). Red-blue and orange-gray color gradient arrows in the figure illustrate low temperature and mechanical shrinking stress stimuli. Scale bar: 250 μm.

Cell attachment and detachment assays were conducted for stretched and unstretched Low-PI-PDMS (Figure 5. (i) and (ii)) using four different protocols, as illustrated in Figure 1. One unstretched and three stretched Low-PI-PDMS surfaces were employed for the cell attachment assay. Since two of the three cell attachment profiles of stretched Low-PI-PDMS surfaces almost overlap, one of the two cell attachment profiles is indicated by blue circles in Figure 5(i). Phase-contrast images of adhered cells on unstretched and stretched Low-PI-PDMS surfaces at 24 h after seeding are shown in Figure 6(A), (C), (E) and (G). The unstretched Low-PI-PDMS surface had 7.4 ± 1.0 (×10^3^) adhered cells/cm^2^ at 24 h after seeding (pink circles in Figure 5(i) and Figure 6(A)), while stretched Low-PI-PDMS surfaces had 5.7 ± 0.9 (×10^3^) adhered cells/cm^2^ (green circles in Figure 5(i) and Figure 6(E)) and 4.6 ± 0.3 (×10^3^) cells/cm^2^ (blue circles in Figure 5(i) and Figure 6(G)). The unstretched Low-PI-PDMS surface was more cell adhesive than stretched Low-PI-PDMS surface. This difference in the number of adhered cells between unstretched and stretched Low-PI-PDMS surfaces is discussed below.

In contrast, a previous report demonstrated that the number of adhered cells on the unstretched and stretched Lar-PI-PDMS surface are 3.0 ± 0.3 (×10^3^) cells/cm^2^ and 3.9 ± 1.2 (×10^3^) cells/cm^2^, respectively, using same cell seeding and culture conditions as Low-PI-PDMS [9]. It was found that, besides stretched Low-PI-PDMS surface (84.7° ± 2.9° (Figure 3), 5.7 ± 0.9 (×10^3^) cells/cm^2^ (green circles in Figure 5(i)) and 4.6 ± 0.3 (×10^3^) cells/cm^2^ (blue circles in Figure 5(i)), larger contact angles of Low-PI-PDMS and Lar-PI-PDMS surfaces were more cell adhesive at 37 °C; unstretched Low-PI-PDMS (79.6° ± 2.8° (Figure 3), 7.4 ± 1.0 (×10^3^) cells/cm^2^ (pink circles in Figure 5(i)) > stretched Lar-PI-PDMS (77.6° ± 1.9°, 3.9 ± 1.2 (×10^3^) cells/cm^2^) > unstretched Lar-PI-PDMS (75.5° ± 1.3°, 3.0 ± 0.3 (×10^3^) cells/cm^2^) as described above. Although the graft density of grafted PIPAAm of stretched and unstretched Low-PIPAAm-PDMS surfaces was compared, there was no difference between these two surfaces. This is similar to previous results showing that the applied stretching stress (SR = 20%) does not affect the graft polymer density of the Lar-PI-PDMS surface, presumably because ATR/FT-IR is not able to detect subtle decreases in the graft polymer density [9]. The cell detachment assay for the Lar-PI-PDMS surface demonstrates that the applied uniaxial mechanical stretching stress not only increases the contact angle of the Lar-PI-PDMS surface but also enhances its cell adhesiveness, because of thinning of the grafted PIPAAm gel layer and dehydration of its polymeric chains [9]. After culturing cells on the stretched Lar-PI-PDMS surface, the dual stimuli (Protocol D in Figure 1) of low temperature and releasing the external stretching stress (shrinking the stretched Lar-PI-PDMS surface) promoted cell detachment from the stretched Lar-PI-PDMS surface. The dual stimuli enabled to faster acceleration of the hydration of the grafted PIPAAm chains compared to a single stimulus, either low temperature treatment or shrinking mechanical stress (Protocol A, B, and C in Figure 1).

Regarding the cell attachment behavior of the stretched Low-PI-PDMS surface, it was initially expected that, since stretched Low-PI-PDMS surfaces exhibited larger contact angles than the unstretched surfaces, the stretched Low-PI-PDMS surfaces would be more cell adhesive. However, contrary to expectations, the unstretched Low-PI-PDMS surface (pink circle in Figure 5(i) was more cell adhesive than the stretched Low-PI-PDMS surface (green and blue circles Figure 5(i)). This cell adhesiveness was different from that of the Lar-PI-PDMS, in which the stretched state showed more cell adhesive than unstretched surface, as mentioned above. To investigate these different cell adhesion behaviors, the amount of adsorbed FN molecules on Lar-PI-PDMS and Low-PI-PDMS surfaces in stretched and unstretched states were examined (Table 1), as FN adsorption experiments were not conducted yet. The external mechanical stretching stress almost doubled the amount of adsorbed FN from 70 ± 20 ng/cm^2^ (unstretched Lar-PI-PDMS) to 160 ± 20 ng/cm^2^ (stretched Lar-PI-PDMS) at 37 °C, while tripling the Low-PI-PDMS from 100 ± 20 ng/cm^2^ (unstretched Low-PI-PDMS) to 290 ± 0 ng/cm^2^ (stretched Low-PI-PDMS) at 37 °C. However, for the PDMS surface, external mechanical stress did not exhibit a significant increase in adsorbed FN at 37 °C and 20 °C. This phenomenon was supported by the equal contact angles of PDMS irrespective of the external mechanical stretching. The remarkable increase in the adsorbed FN molecules was due to enhancement of the hydrophobic properties of the Lar-PI-PDMS and Low-PI-PDMS surfaces with external mechanical stretching stress, as described above. The external mechanical stress rendered the stretched Lar-PI-PDMS surface more FN adhesive than unstretched Lar-PI-PDMS surface. This result strongly supports the previous result that the stretched Lar-PI-PDMS surface is more cell adhesive than the unstretched PI-PDMS one [9].

**Table 1:** Density of adsorbed fibronectin on stretched and unstretched PDMS, Low-PI-PDMS and Lar-PI-PDMS surfaces.

	20 °C		37 °C	
Stretch ratio (%)	0%	20%	0%	20%
PDMS∗	140 ± 20	170 ± 20	160 ± 40	200 ± 10
Low-PI-PDMS∗	ND		100 ± 20	290 ± 0
Lar-PI-PDMS∗	ND	30 ± 20	70 ± 20	160 ± 20

∗Unit: ng/cm^2^, ND = Not detected.

In contrast, the stretched Low-PI-PDMS showed stronger hydrophobic properties and a larger amount of adsorbed FN than the unstretched Low-PI-PDMS surfaces. However, the cell adhesive property of the unstretched Low-PI-PDMS surface was superior to that of the stretched Low-PI-PDMS surface. This difference was probably due to denaturation of adsorbed FN on the strongly hydrophobic stretched Low-PI-PDMS surface, as described below.

At 20 °C, the adsorbed FN molecules of the Low-PI-PDMS and Lar-PI-PDMS surfaces were not detected or extremely decreased compared to 37 °C. However, irrespective of the external mechanical stretching, the PDMS surfaces did not exhibit s significant difference in FN adsorption between 20 °C and 37 °C. The reduction of adsorbed FN demonstrated hydration of grafted PIPAAm chains with low temperature treatment.

The conformation of adsorbed FN affects subsequent cell adhesion as the cell-binding domain in FN is exposed to interact with α5β1 integrin, when FN molecules are adsorbed on an appropriate hydrophobic surface, such as a TCPS surface [26, 27, 28], with contact angle of 62.8° ± 1.6° (37 °C). As a result, cells are favorably able to adhere to and spread on the FN adsorbed TCPS surface. However, bacteriological grade polystyrene surface (BPS) (contact angle = 72.5° ± 1.4° (37 °C), which is more hydrophobic than TCPS, denatures the adsorbed FN molecules such that the cell-binding domain is not accessible to α5β1 integrin, even though a large amount of FN molecules are adsorbed on a BPS [26]. Considering the increased hydrophobicity and increased amount of adsorbed FN on the stretched Low-PI-PDMS surface, denaturation of adsorbed FN could be induced, resulting in less cell adhesiveness than that of the unstretched Low-PI-PDMS surface.

The cell detachment assay was conducted for stretched and unstretched Low-PI-PDMS surfaces after 24 h cell culture using four different protocols, Protocols A, B, C and D illustrated in Figure 1 (Figure 5(ii) and Figure 6). Only the shrinking mechanical stress stimulus does not induce cell detachment (yellow circles in Figure 5(ii), Figure 6(D) (Protocol B)), which is consistent with incomplete cell detachment from the stretched Lar-PI-PDMS surfaces, as previously reported [9]. Sufficient hydration on the grafted PIPAAm chains of Low-PI-PDMS surface was not induced for subsequent cell detachment with only external mechanical shrinking. A low temperature stimulus almost completely detached cells from unstretched Low-PI-PDMS surfaces (pink circles in Figure 5(ii), Figure 6(B) (Protocol A)), whereas a low temperature stimulus slowly detached cells from stretched Low-PI-PDMS surfaces where adsorbed FN was denatured (green circles in Figure 5(ii), and Figure 6(F) (Protocol C)). The low temperature stimulus hydrated the PIPAAm chains of the unstretched and stretched Low-PI-PDMS surfaces, altering their surface to be hydrophilic, as indicated by the contact angles (Figure 3). However, the denatured FN could be strongly adsorbed on the stretched Low-PI-PDMS surface, possibly suppressing cell detachment. Therefore, adhered cells remained on the stretched Low-PI-PDMS surfaces, even after 120 min (green circles in Figure 5(ii), and Figure 6(F)). The dual stimuli, low temperature followed by external mechanical shrinking (blue circles in Figure 5(ii), Figure 6(H) (Protocol D)), resulted in nearly complete cell detachment from stretched Low-PI-PDMS within 90 min; cells were completely detached in 120 min. Complete detachment from the stretched Low-PI-PDMS surfaces with dual stimuli is consistent with that from the stretched Lar-PI-PDMS surfaces, as mentioned above [9]. Presumably, considering these results, even though adsorbed FN were denatured, the dual stimuli of low temperature and external mechanical stress efficiently promoted hydration of grafted PIPAAm chains and detachment of adhered cells. Mechanical stress-induced dehydration markedly promoted when the grafted PIPAAm gel layer was extremely thin. Although a previous report demonstrated that density of grafted polymer chains decreased with the applied mechanical stress [29], it was not evident that applied mechanical stress decreased the graft polymer density in this study. The author believes that the applied mechanical stress would decrease the graft polymer density, as well as various surface properties, such as wettability (contact angles), cell adhesiveness, and molecular mobility of the grafted polymer chains.

## 4 Conclusions

The long-term contact angle measurements (61 days) demonstrated that the grafted PIPAAm chains of Lar-PI-PDMS and Low-PI-PDMS surfaces maintained the properties of temperature-dependent hydration and dehydration without suffering hydrophobic recovery of the PDMS surface. This suggested that the cross-linked structure of the grafted PIPAAm gel suppressed the transport of low-molecular weight of siloxane to the PDMS surface. FN adsorption experiments revealed that external mechanical stretching stress on the Lar-PI-PDMS surface increased the amount of adsorbed FN, resulting in enhanced cell adhesion properties. Although external mechanical stretching also enhanced the hydrophobicity of the Low-PI-PDMS surface and increased the amount of adsorbed FN, the number of adhered cells on the stretched Low-PI-PDMS surface was less than that of the unstretched Low-PI-PDMS surface, probably because of denaturation of adsorbed FN on the more hydrophobic surface of the stretched Low-PI-PDMS. Additionally, mechanical stretching stress extensively promoted the dehydration of grafted PIPAAm chains when the thickness of the grafted PIPAAm gel was thin. The dual stimuli of low temperature treatment and mechanical shrinking stress efficiently promoted the dehydration of grafted PIPAAm chains, even though the thickness of grafted PIPAAm layer were thin. Especially, the applied mechanical stretching highly enhanced hydrophobicity of PI-PDMS surfaces such that adsorbed proteins were denatured, when the grafted PIPAAm layer was extremely thin as Low-PI-PDMS surface. These results suggested that the PIPAAm-grafted PDMS was stably utilized as a stretchable temperature-responsive cell culture surface without significant hydrophobic recovery of basal PDMS. External mechanical stress affected properties of the PIPAAm-grafted surface, depending on the graft-polymer thickness. EB irradiation may be a useful method for preparing chemically stable polymeric-gel-grafted PDMS surfaces, suppressing hydrophobic recovery. The stretch ratio of the PDMS substrate used in this study was limited because the maximum stretch ratio was approximately 25%. Therefore, a higher stretchable ratio was not employed for the PI-PDMS surface. Recently, highly stretchable PDMS has been developed, being uniaxially stretched to 7-fold in length with external mechanical stress [30]. In the future, using such stretchable PDMS as basal substrate, a highly stretchable PIPAAm-grafted PDMS surface may be prepared, possibly providing a wide range of surface hydrophilic and hydrophobic properties with dual stimuli, such as temperature and external mechanical stress, without optimizing the thickness and/or density of the grafted PIPAAm layer.

## Declarations

### Author contribution statement

Yoshikatsu Akiyama: Conceived and designed the experiments; Performed the experiments; Analyzed and interpreted the data; Contributed reagents, materials, analysis tools or data; Wrote the paper.

### Funding statement

This work was supported by Grant-in-aid for Scientific Research, KAKENHI (18K12084).

### Data availability statement

Data will be made available on request.

### Declaration of interests statement

The authors declare no conflict of interest.

### Additional information

No additional information is available for this paper.
